# Early Detection of Lumpy Skin Disease in Cattle Using Deep Learning—A Comparative Analysis of Pretrained Models

**DOI:** 10.3390/vetsci11100510

**Published:** 2024-10-17

**Authors:** Chamirti Senthilkumar, Sindhu C, G. Vadivu, Suresh Neethirajan

**Affiliations:** 1 Department of Computing Technologies, School of Computing, SRM Institute of Science and Technology, Kattankulathur 603203, India; chamirtinms@gmail.com (C.S.); sindhucmaa@gmail.com (S.C.); 2 Department of Data Science and Business Systems, School of Computing, SRM Institute of Science and Technology, Kattankulathur 603203, India; vadivug@srmist.edu.in; 3 Department of Animal Science and Aquaculture, Faculty of Agriculture, Dalhousie University, P.O. Box 550, Truro, NS B2N 5E3, Canada; 4 Faculty of Computer Science, Dalhousie University, 6050 University Ave, Halifax, NS B3H 1W5, Canada

**Keywords:** lumpy skin disease, deep learning, automated disease detection, veterinary diagnostics, artificial intelligence, bovine health management, digital livestock farming

## Abstract

Lumpy Skin Disease (LSD) poses a significant threat to agricultural economies, particularly in livestock-dependent countries like India, due to its high transmission rate leading to severe morbidity and mortality among cattle. This underscores the urgent need for early and accurate detection to effectively manage and mitigate outbreaks. Leveraging advancements in computer vision and artificial intelligence, our research develops an automated system for LSD detection in cattle using deep learning techniques. We utilized two publicly available datasets comprising images of healthy cattle and those with LSD, including additional images of cattle affected by other diseases to enhance specificity and ensure the model detects LSD specifically rather than general illness signs. Our methodology involved preprocessing the images, applying data augmentation, and balancing the datasets to improve model generalizability. We evaluated over ten pretrained deep learning models—Xception, VGG16, VGG19, ResNet152V2, InceptionV3, MobileNetV2, DenseNet201, NASNetMobile, NASNetLarge, and EfficientNetV2S—using transfer learning. The models were rigorously trained and tested under diverse conditions, with performance assessed using metrics such as accuracy, sensitivity, specificity, precision, F1-score, and AUC-ROC. Notably, VGG16 and MobileNetV2 emerged as the most effective, achieving accuracies of 96.07% and 96.39%, sensitivities of 93.75% and 98.57%, and specificities of 97.14% and 94.59%, respectively. Our study critically highlights the strengths and limitations of each model, demonstrating that while high accuracy is achievable, sensitivity and specificity are crucial for clinical applicability. By meticulously detailing the performance characteristics and including images of cattle with other diseases, we ensured the robustness and reliability of the models. This comprehensive comparative analysis enriches our understanding of deep learning applications in veterinary diagnostics and makes a substantial contribution to the field of automated disease recognition in livestock farming. Our findings suggest that adopting such AI-driven diagnostic tools can enhance the early detection and control of LSD, ultimately benefiting animal health and the agricultural economy.

## Simple Summary

Lumpy Skin Disease (LSD) is a highly contagious viral infection in cattle that poses a significant threat to agricultural economies, especially in countries like India. Early and accurate detection is vital to prevent widespread outbreaks and reduce economic losses. Our research leverages advancements in artificial intelligence (AI), specifically deep learning, to develop an automated system for detecting LSD in cattle. We utilized publicly available datasets containing images of healthy cattle, those affected by LSD, and, importantly, cattle with other skin diseases to ensure our model specifically identifies LSD rather than general illness signs. We evaluated over ten pretrained deep learning models, including VGG16 and MobileNetV2, using transfer learning techniques. Through detailed data preprocessing, augmentation, and balancing, we enhanced model performance and generalizability. We assessed the models using crucial medical diagnostics metrics like sensitivity and specificity to minimize false negatives and positives. VGG16 and MobileNetV2 stood out, achieving high accuracy along with excellent sensitivity and specificity, effectively detecting LSD without misclassifying other conditions. This study provides valuable insights into applying deep learning in veterinary diagnostics, contributing to developing reliable AI tools for early LSD detection, ultimately improving animal health management and safeguarding the agricultural economy.

## 1. Introduction

Lumpy Skin Disease (LSD) is a significant transboundary viral disease primarily affecting bovines such as cattle and water buffaloes. First identified in Zambia, Southern Africa, in 1929, it was initially mistaken for conditions like plant poisoning or insect stings [1]. By 1943, LSD had spread to Botswana and rapidly overtook the northwestern region of Africa, infecting up to 8 million cattle. The disease continued its spread, reaching Israel by 1989 [2] and subsequently affecting several Middle Eastern countries. Outbreaks occurred in Jordan, Iraq, and Turkey in 2013, followed by Russia’s first outbreak in 2015 [3]. By 2019, LSD had reached Asia, with India reporting its first outbreak in Odisha in August 2019, followed by a more severe outbreak in 2022 [4].

**Figure 1:**
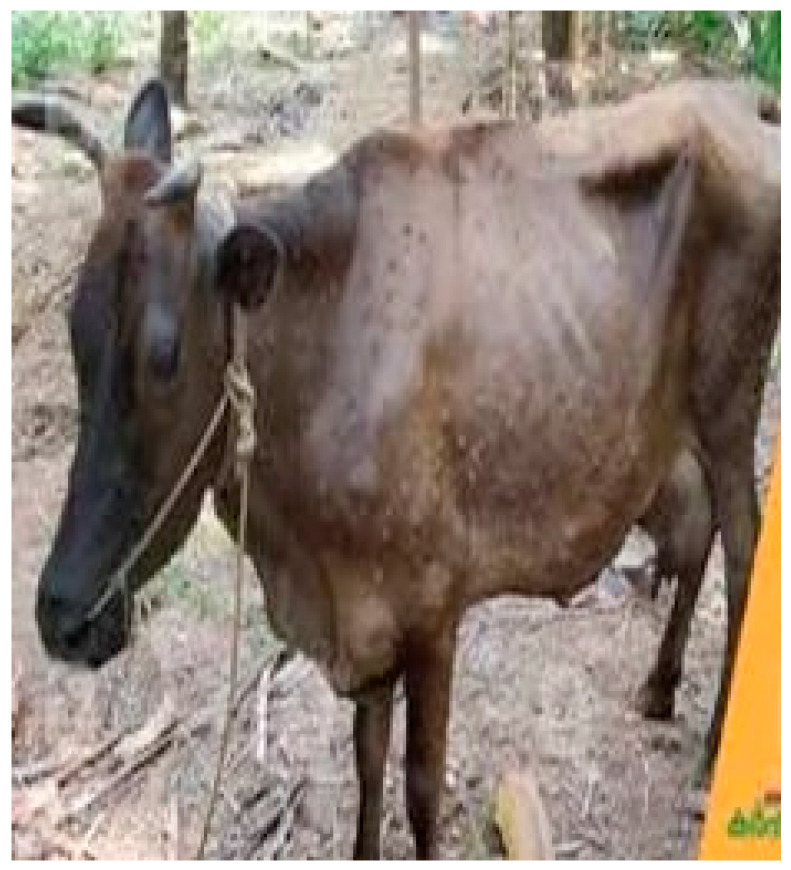
A cow exhibiting characteristic symptoms of Lumpy Skin Disease (LSD), including raised nodules on the skin. These visible signs are critical for early detection and diagnosis, which is further enhanced by the application of deep learning models in this study. The automated detection of such lesions through advanced image analysis techniques can significantly improve the accuracy and speed of LSD identification in bovine populations.

LSD is caused by the Lumpy Skin Disease Virus (LSDV), a member of the Capripoxvirus genus within the Poxviridae family [5,6]. The virus spreads through blood-feeding insects like ticks and mosquitoes or direct contact with infected animals or contaminated objects [7]. Clinically, the disease is characterized by large skin nodules (Figure 1) up to 5 cm in diameter [8], accompanied by symptoms such as fever, reduced milk production, decreased appetite, lameness, and leg edema [9,10]. Laboratory methods like polymerase chain reaction (PCR) and virus isolation are commonly used to identify LSDV [11]. Currently, there is no specific antiviral treatment for LSD; affected cattle receive supportive care, including antibiotics, analgesics, and wound care sprays. Vaccines such as the Neethling vaccine, Kenyan sheep and goat pox (KSGP) O-180 strain vaccines, and Gorgan goat pox (GTP) vaccines are available to prevent the disease’s spread.

India has the world’s largest bovine population, exceeding 300 million, including cows, water buffaloes, and yaks. Dairy production is crucial to the Indian economy, contributing 5% to the nation’s GDP and providing employment to more than 80 million farmers. India also leads globally in milk production, accounting for 24.64% of the world’s total output. However, cattle in India are susceptible to several major diseases, including anthrax, bovine viral diarrhea, bovine tuberculosis, foot and mouth disease, bovine mastitis, papillomatosis, photosensitization, LSD, and infectious bovine keratoconjunctivitis [12,13]. Among these, LSD is particularly dangerous and is classified as a notifiable transboundary viral disease by the World Organization for Animal Health (WOAH) [14]. An LSD outbreak in India could halt cattle exports, leading to significant economic losses and severely affecting rural communities reliant on cattle and agriculture. Integrating deep learning techniques into veterinary diagnostics can enhance the country’s preparedness for major outbreaks like LSD.

Early detection of LSD is critical for reducing morbidity and preventing widespread outbreaks among cattle populations. Farmers often resort to traditional remedies, such as burning neem leaves or disinfecting farms, before consulting a veterinarian. Traditionally, veterinarians perform manual visual inspections, which can be subjective, time-consuming, and prone to errors [15]. By the time the disease is identified, it may have already advanced and spread to other cattle. However, advancements in deep learning models and convolutional neural networks now enable the analysis of cattle skin images to detect even subtle changes indicative of LSD. The availability of datasets containing images of cattle affected by LSD has increased, facilitating the development of automated detection systems. This technology allows farmers and veterinarians to quickly identify the disease and initiate treatment, thereby reducing its spread [16].

The integration of artificial intelligence (AI) and machine learning (ML) into agriculture and veterinary science has significantly advanced livestock health management. Deep learning algorithms are increasingly utilized in areas such as automated cattle recognition, disease detection, behavior analysis, and body condition scoring, all contributing to improved cattle health monitoring [17]. Machine learning models have shown promise in predicting LSD outbreaks. Various models, including logistic regression, support vector machines, decision trees, random forests, and artificial neural networks, have been applied to predict LSD occurrences based on geospatial and meteorological data [18]. Among these, artificial neural networks demonstrated superior performance, achieving an area under the curve (AUC) of 97% and an F1-score of 94%, highlighting their potential in epidemiological forecasting. Optimizing the random forest algorithm with a genetic algorithm for hyperparameter tuning and using the Synthetic Minority Oversampling Technique (SMOTE) to balance datasets has further enhanced prediction accuracy, with improvements of 0.09 in F1-score and 0.04 in AUC [19]. This approach emphasizes the importance of algorithmic fine-tuning and data preprocessing in developing effective predictive models.

Multiple machine learning algorithms have been investigated for predicting LSD outbreaks, with random forest models consistently achieving the highest accuracy. For example, when applied to diverse meteorological and geospatial features, random forest models reached accuracy rates of up to 97.10% [20], reinforcing their robustness in handling complex datasets. Support vector classifiers have also been utilized to forecast LSD outbreaks, analyzing 24,803 cases with 19 predictive variables—including temporal trends and seasonal impacts—and achieving an accuracy of 96.7% in predicting disease spread [21].

Deep learning models have been effectively employed in detecting LSD through image analysis. Preprocessing techniques such as Gaussian filtering and histogram equalization, followed by feature extraction using convolutional neural networks (CNNs), have enabled the classification of cattle images into lumpy skin and normal skin categories with an accuracy of 93% [22]. Further enhancements were achieved using pretrained deep learning models like InceptionV3, ResNet50V2, and VGG16, combined with a modified CNN, yielding accuracies as high as 99.01% on training data and 97.87% on testing data [23]. However, these studies often faced challenges due to small datasets, raising concerns about the generalizability of the models. To address this issue, the current research tests models on larger and more diverse datasets, enhancing robustness.

An innovative approach combining features from ResNet50 and VGG19 through feature fusion and Principal Component Analysis (PCA) achieved a high classification accuracy of up to 99% [24]. Nonetheless, focusing solely on accuracy may not provide a complete understanding of model performance. By incorporating multiple evaluation metrics such as precision, recall, F1-score, specificity, and AUC-ROC, a more comprehensive assessment is offered. Lastly, the DenseNet121 model, applied to a balanced dataset of 4759 images, achieved an accuracy of 99.10%, outperforming several other models [25]. Despite these high accuracies, expanding the range of deep learning models evaluated could provide a more thorough understanding of their relative performance in LSD detection.

In this study, we implemented and evaluated a range of pretrained deep learning models for the detection of Lumpy Skin Disease (LSD) in cattle. Our objective was to identify the most accurate and reliable models for classifying cattle images as either normal or affected by LSD. We introduced the application of several advanced deep learning models that are less commonly utilized in LSD research. A thorough comparative analysis was performed using multiple evaluation metrics, providing valuable insights for future research in selecting the most suitable models for LSD detection. Additionally, we explored various hyperparameters, including learning rates and optimizers, to optimize the models’ performance.

## 2. Materials and Methods

### 2.1. Data Acquisition

The foundation of this study lies in the acquisition of high-quality images of cattle, both healthy and those diagnosed with Lumpy Skin Disease (LSD). Two distinct datasets were utilized to enhance the model’s generalizability and mitigate the risk of overfitting. The first dataset [26] was sourced from the Mendeley Data repository and comprises 1024 images, including 700 images of healthy cows and 324 images of cows affected by LSD. The images in this dataset were preprocessed and uniformly resized to 256 × 256 pixels in PNG format. This dataset is diverse, featuring various breeds of bovine cattle such as Santa Gertrudis, American Brahmans, Guzerats, and Yaks, with images taken from different orientations, positions, and lighting conditions. The second dataset [27], consisting of 2223 images (1520 healthy and 703 LSD-affected), was also preprocessed, with all images resized to 640 × 640 pixels. To further enhance diversity and robustness, this dataset underwent data augmentation. Augmentations applied include horizontal and vertical flips, 90-degree rotations, random cropping, and shear transformations. These augmentations help simulate real-world variations in image data, providing the model with a more comprehensive learning environment.

### 2.2. Image Preprocessing

To ensure consistency across the datasets, all images were scaled to a standardized size of 256 × 256 pixels. This uniformity is crucial for feeding the images into the model’s input layer, facilitating efficient learning. The datasets were then split into training and validation sets with a 90–10 split, respectively. The training set was employed during the model learning stage, while the validation set was used to assess the model’s performance and prevent overfitting. Further image augmentation techniques were applied to the training data to improve the model’s robustness. Techniques such as horizontal and vertical flips, shear range adjustments, zoom adjustments, and rotations were employed. These augmentations help the model generalize better by simulating the variability found in real-world data, ultimately leading to improved accuracy during validation.

### 2.3. Model Selection and Transfer Learning

Given the relatively small size of the datasets, transfer learning was employed as the primary method for developing the deep learning model. Transfer learning allows the reuse of pre-trained models—networks initially trained on large datasets for other tasks—by fine-tuning them for the specific task of LSD detection. This method is particularly effective when working with limited data, as it leverages the low-level feature extraction capabilities of pre-trained models. The Keras library offers a range of pre-trained models, and ten different models were selected for comparison in this study: Xception, VGG16, VGG19, ResNet152V2, InceptionV3, InceptionResNetV2, MobileNetV2, DenseNet201, NASNetLarge, and EfficientNetV2S. Each model was chosen based on its proven effectiveness in image classification tasks, particularly in medical and veterinary fields.

### 2.4. Model Development

The pre-trained models were initialized with weights from the ImageNet dataset, a vast image dataset containing over 14 million annotated images. These weights serve as a starting point, allowing the models to begin with already established feature extraction capabilities. The layers of the pre-trained models were frozen to prevent overwriting the pre-learned feature extraction techniques during the initial stages of training. Additional layers specific to LSD detection were then stacked on top of the base models. The sequential model in Keras was utilized for this purpose, where the output from each layer is fed as input to the next layer. This process continues until the final output layer is reached. The activation functions used include the Rectified Linear Unit (ReLU) for intermediate layers and the Softmax function for the final classification layer. ReLU was chosen for its efficiency in handling positive inputs, while Softmax was used to produce a probability distribution over the two classes (normal skin and lumpy skin), making it ideal for the classification task.

### 2.5. Hyperparameter Tuning

To optimize the performance of the deep learning models, we conducted comprehensive hyperparameter tuning. This process involved experimenting with various configurations to determine the optimal settings for our specific datasets and models. We tested two learning rates (0.001 and 0.0001) and two optimization algorithms: Adam and Stochastic Gradient Descent (SGD). The Adam optimizer consistently outperformed SGD across all models and datasets, achieving higher accuracies. Specifically, models trained with Adam reached an accuracy of up to 92%, whereas those trained with SGD achieved a maximum accuracy of 67%. The superior performance of Adam can be attributed to its adaptive learning rate and momentum, which help in navigating the loss landscape more effectively than the fixed learning rate of SGD. Regarding the learning rate, a value of 0.001 was found to be optimal for most models. This learning rate provided a good balance between convergence speed and model performance. When we reduced the learning rate to 0.0001, there was no significant improvement in accuracy, and the training time increased substantially due to slower convergence. Therefore, we selected a learning rate of 0.001 for the final models.

We also experimented with different batch sizes and training epochs to assess their impact on model performance and computational efficiency. Batch sizes of 32 and 64 were tested alongside 10 and 15 training epochs. A batch size of 32 with 10 epochs yielded the best results in terms of both accuracy and training time. This configuration allowed the models to learn effectively without overfitting, as increasing the number of epochs did not result in significant accuracy gains but did increase the risk of overfitting. The model complexity varied among the different architectures, reflected in the total number of parameters. For example, the VGG16 model on Dataset 1 had approximately 15,242,050 trainable parameters. In contrast, the MobileNetV2 model on Dataset 2 had around 3,571,778 parameters. The substantial difference in parameter counts highlights the trade-off between model complexity and computational efficiency. MobileNetV2, being a more lightweight model, is advantageous for deployment in resource-constrained environments without significantly compromising accuracy.

### 2.6. Model Evaluation

The performance of each model was evaluated using a range of metrics: accuracy, precision, recall, F1-score, specificity, and the area under the Receiver Operating Characteristic (ROC) curve (AUC-ROC). These metrics were calculated based on the true positive, true negative, false positive, and false negative values derived from the model’s predictions. To visualize the model’s performance during training, accuracy and loss curves were plotted for both the training and validation sets. These plots helped in identifying signs of overfitting, such as increasing training accuracy accompanied by decreasing validation accuracy, or decreasing training loss coupled with increasing validation loss. Additionally, confusion matrices were generated to provide a more detailed view of the model’s classification performance, and ROC curves were plotted to assess the trade-off between true positive rates and false positive rates. The AUC-ROC value was used as a comprehensive measure of the model’s performance, with higher values indicating better discriminatory capability.

### 2.7. Transfer Learning Models and Testing

The models were tested on both datasets, with each model’s performance evaluated using the aforementioned metrics. The results provide valuable insights into the effectiveness of various pre-trained models for LSD detection and highlight the strengths and weaknesses of each approach. The findings will guide future research in selecting the most appropriate base models and hyperparameters for similar veterinary diagnostic tasks.

### 2.8. Experimental Setup

The experiments were conducted on a computing system equipped with an 11th Generation Intel^®^ Core™ i5-1135G7 CPU (Santa Clara, CA, USA) operating at 2.40 GHz and 8 GB of RAM. For graphical computations, an NVIDIA GeForce MX330 GPU with 128 MB of VRAM was utilized. Although the GPU had limited video memory, it provided sufficient acceleration for training the models given the optimized configurations. The software environment was established using Anaconda, facilitating package management and deployment. The deep learning models were implemented using Python 3.12.3 with the TensorFlow 2.17.0 and Keras 3.4.1 libraries, which are well-suited for developing and training deep neural networks. All coding and experimentation were performed within a Jupyter Notebook 7.0.8 environment, allowing for interactive development and immediate visualization of the results.

## 3. Results

**Figure 2:**
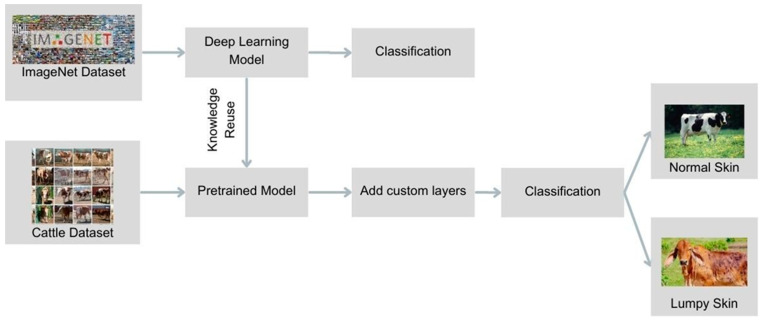
Working principle of transfer learning in Lumpy Skin Disease detection.

Transfer learning, a subset of deep learning, involves repurposing knowledge gained from one task to another, making it particularly effective when working with limited data. The initial layers of a deep learning model typically focus on identifying low-level features such as corners, edges, and colors. By using a pre-trained model, these basic features were extracted and then tailored to the specific task of LSD detection by adding new layers (Figure 2).

### 3.1. Xception Model Performance

**Figure 3:**
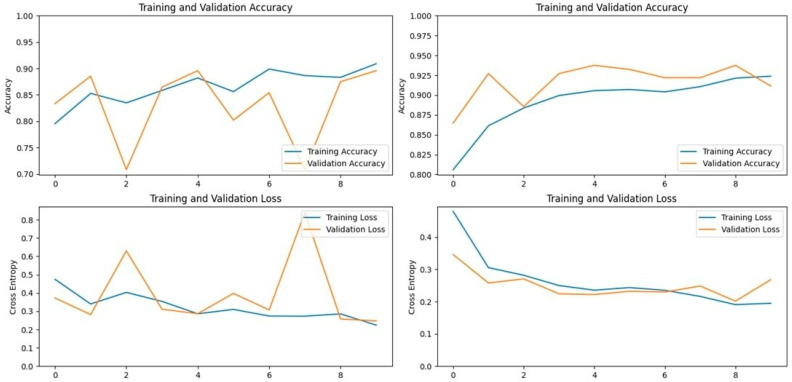
Accuracy and loss graphs for the Xception model on Dataset 1 (**left**) and Dataset 2 (**right**).

**Figure 4:**
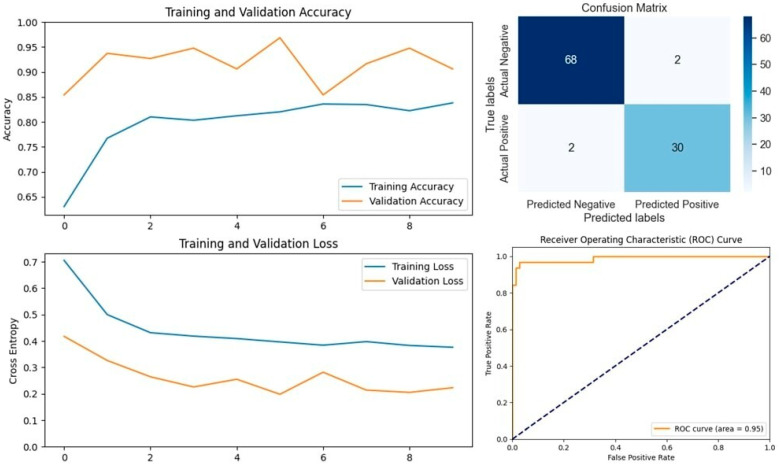
Performance metrics for the VGG16 model on Dataset 1, including accuracy, loss, confusion matrix, and ROC curve.

The Xception model, which stands for Extreme Inception, is a deep convolutional neural network with 71 layers, developed by Google researchers. It replaces standard inception modules with depth-wise separable convolutions, allowing for efficient feature extraction from images. The pre-trained Xception model, originally trained on the ImageNet database, was able to classify a wide range of image categories, which made it a strong candidate for detecting LSD. When applied to our datasets, the Xception model achieved an accuracy of 90.19% on the first dataset and 93.24% on the second dataset, demonstrating its capability in distinguishing between healthy and affected cattle (Figure 3 and Figure 4).

### 3.2. VGG16 and VGG19 Models

**Figure 5:**
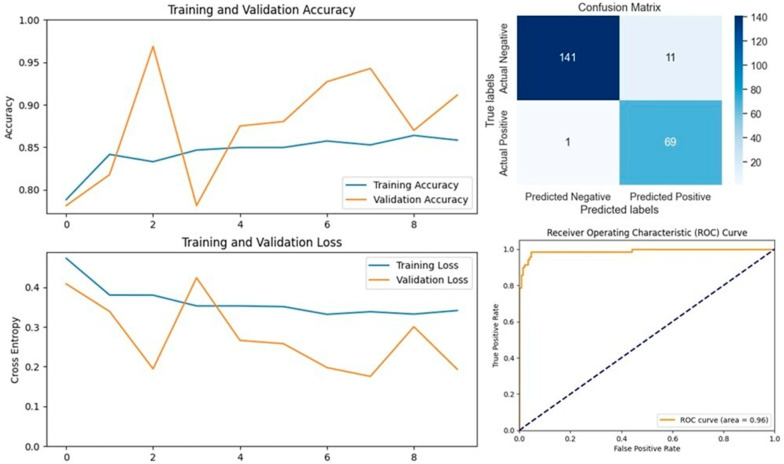
Performance metrics for the VGG16 model on Dataset 2, including accuracy, loss, confusion matrix, and ROC curve.

**Figure 6:**
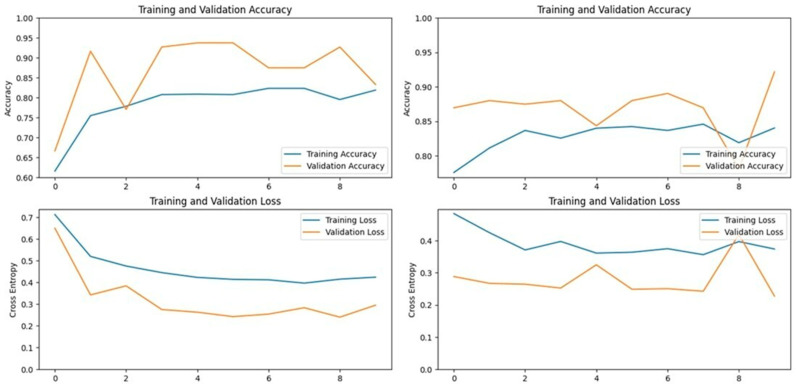
Accuracy and loss graphs for the VGG19 model on Dataset 1 (**left**) and Dataset 2 (**right**).

**Figure 7:**
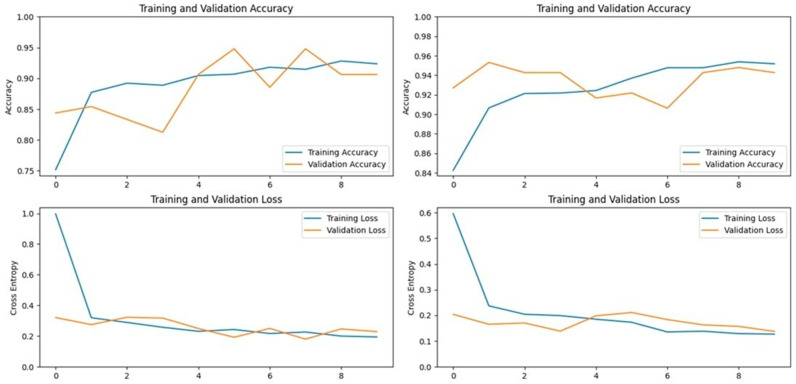
Accuracy and loss graphs for the ResNet152V2 model on Dataset 1 (**left**) and Dataset 2 (**right**).

**Figure 8:**
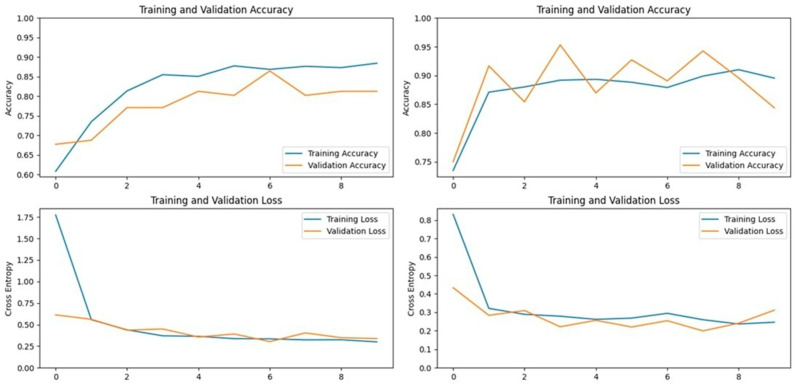
Accuracy and loss graphs for the InceptionV3 model on Dataset 1 (**left**) and Dataset 2 (**right**).

The VGG16 model, known for its simplicity and depth, consists of 16 layers, including 13 convolutional layers and 3 fully connected layers. This structure allows it to progressively extract features from images, making it highly effective in image classification tasks. In our study, VGG16 outperformed other models, achieving an accuracy of 96.07% on the first dataset and 94.59% on the second dataset, making it the best-performing model for the first dataset (Figure 5 and Figure 6). Similarly, the VGG19 model, which extends VGG16 by adding three more convolutional layers, performed well in detecting LSD. VGG19 achieved accuracies of 94.11% and 92.79% on the first and second datasets, respectively. These results underscore the effectiveness of the VGG architecture in feature extraction and classification tasks related to LSD (Figure 7 and Figure 8).

### 3.3. ResNet152V2 Model

**Figure 9:**
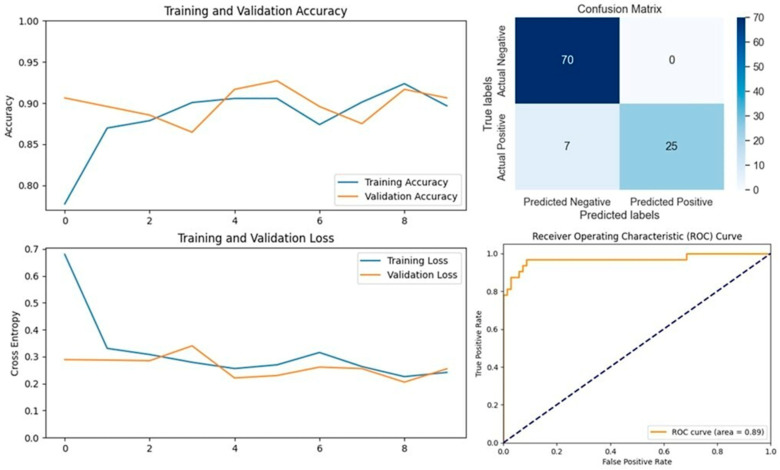
Performance metrics for the MobileNetV2 model on Dataset 1, including accuracy, loss, confusion matrix, and ROC curve.

**Figure 10:**
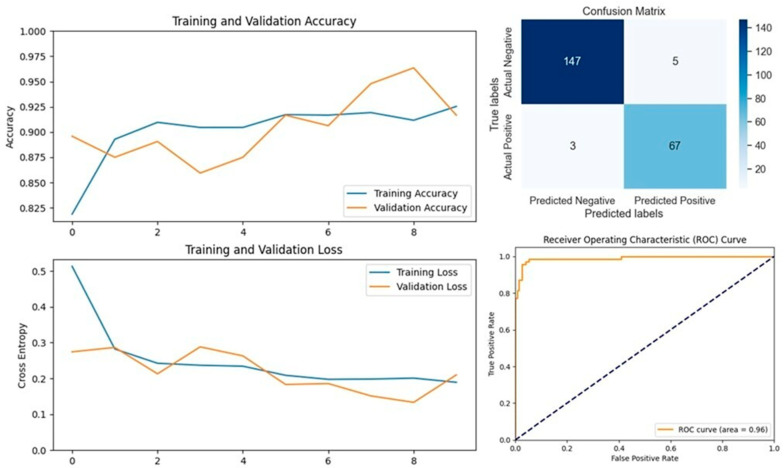
Performance metrics for the MobileNetV2 model on Dataset 2, including accuracy, loss, confusion matrix, and ROC curve.

ResNet152V2, a 152-layer deep neural network, was designed to address the vanishing gradient problem, which often hinders the performance of deep models. This model was selected for its high top-1 and top-5 accuracies on the ImageNet dataset, which were 78% and 94.2%, respectively. In our study, ResNet152V2 achieved an accuracy of 95.09% on the first dataset and 95.94% on the second dataset, indicating its robustness in handling complex image classification tasks (Figure 9 and Figure 10).

### 3.4. InceptionV3 Model

**Figure 11:**
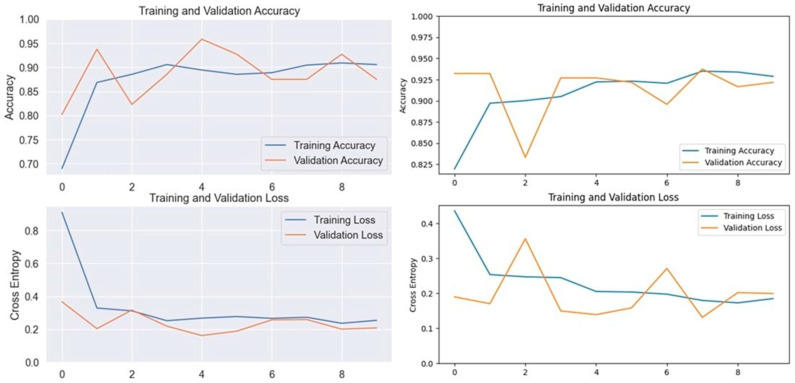
Accuracy and loss graphs for the DenseNet201 model on Dataset 1 (**left**) and Dataset 2 (**right**).

**Figure 12:**
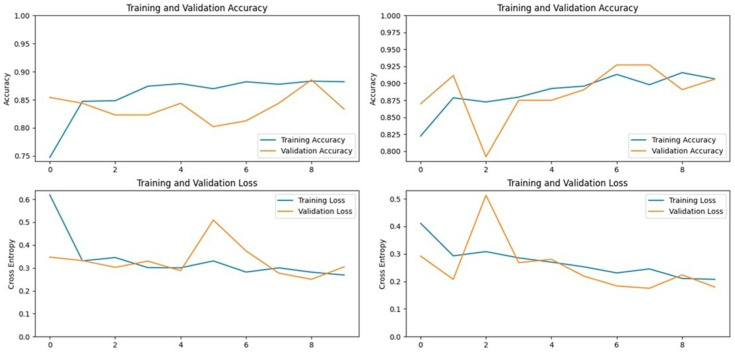
Accuracy and loss graphs for the NASNetMobile model on Dataset 1 (**left**) and Dataset 2 (**right**).

The InceptionV3 model is a deep convolutional neural network that extracts general features from images before classifying them. Trained on over 14 million images from the ImageNet dataset, it has learned to identify important features across various animal classes, making it suitable for LSD detection. In our application, InceptionV3 achieved an accuracy of 87.25% on the first dataset and 95.94% on the second dataset, demonstrating its effectiveness in certain scenarios but also indicating potential variability depending on the dataset used (Figure 11 and Figure 12).

### 3.5. MobileNetV2 Model

**Figure 13:**
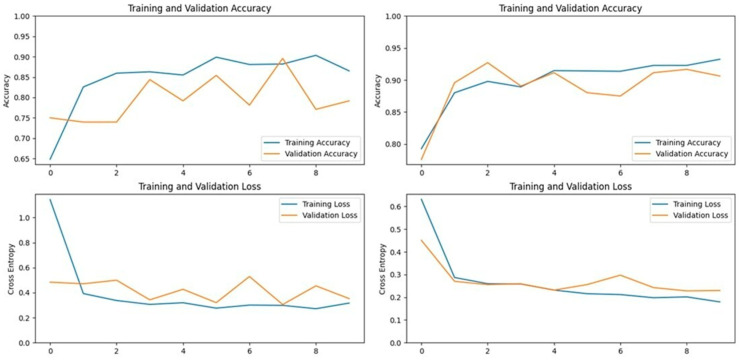
Accuracy and loss graphs for the NASNetLarge model on Dataset 1 (**left**) and Dataset 2 (**right**).

**Figure 14:**
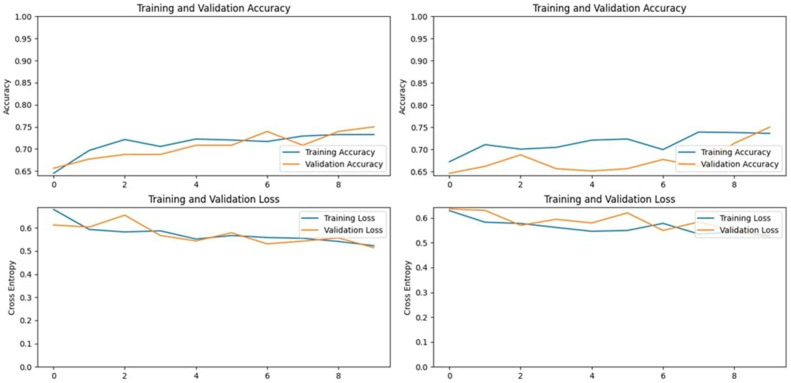
Accuracy and loss graphs for the EfficientNetV2S model on Dataset 1 (**left**) and Dataset 2 (**right**).

MobileNetV2, specifically designed for mobile applications, is a 53-layer deep convolutional neural network that includes depth-wise separable convolutions and linear bottlenecks. This model was particularly effective in our study, achieving accuracies of 93.13% on the first dataset and 96.39% on the second dataset. MobileNetV2 not only performed well but also demonstrated the best accuracy among all tested models on the second dataset, highlighting its potential for efficient and accurate LSD detection in resource-constrained environments (Figure 13 and Figure 14).

### 3.6. DenseNet201 Model

**Figure 15:**
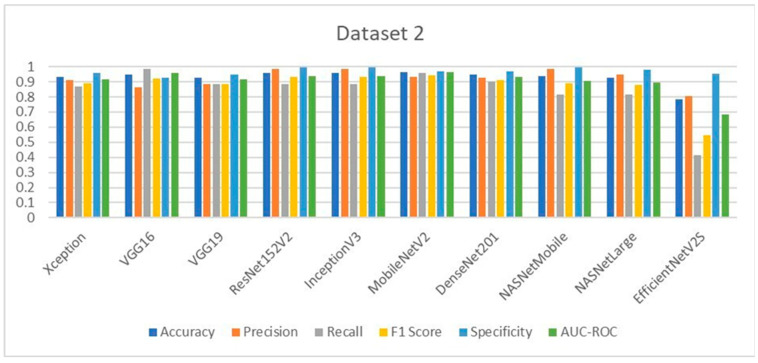
Comparative performance analysis of all 10 models on Dataset 1.

**Figure 16:**
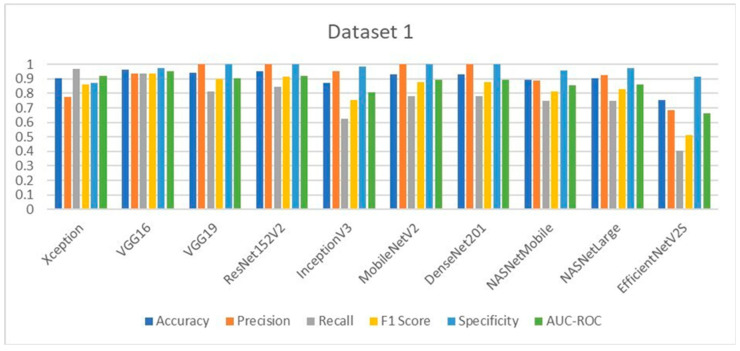
Comparative performance analysis of all 10 models on Dataset 2.

DenseNet201 is another deep convolutional neural network that uses dense connectivity between layers, which helps in feature reuse and boosts the model’s performance. This model, with its 201 layers, performed admirably in our study, achieving accuracies of 93.13% on the first dataset and 94.59% on the second dataset. DenseNet201’s ability to maintain high performance across different datasets suggests its reliability in various conditions (Figure 15 and Figure 16).

### 3.7. NASNet Models (NASNetMobile and NASNetLarge)

NASNet is a deep convolutional neural network developed by Google, which uses Neural Architecture Search (NAS) to optimize the search for the best-performing neural network architecture. NASNetMobile, a more compact version, achieved accuracies of 89.21% on the first dataset and 93.69% on the second dataset. Although NASNetMobile performed well, it did not surpass the top performers in our study. On the other hand, NASNetLarge, a larger version of the NASNet model with over 80 million parameters, achieved accuracies of 90.19% on the first dataset and 92.79% on the second dataset. While NASNetLarge did not outperform the best models, it provided valuable insights into the trade-offs between model complexity and performance in LSD detection.

### 3.8. EfficientNetV2S Model

EfficientNetV2S, another deep convolutional neural network architecture, also operates based on Neural Architecture Search. Despite performing well on the ImageNet dataset, EfficientNetV2S did not achieve the same level of success in detecting LSD in cattle. It attained the lowest accuracy among all tested models, with 75.49% on the first dataset and 78.37% on the second dataset. However, in terms of specificity, it did manage to outperform some models on certain metrics, indicating that while it may not be the best for overall accuracy, it could be valuable in specific scenarios.

### 3.9. Comparative Analysis of Deep Learning Models for LSD Detection

The performance of the ten deep learning models deployed for Lumpy Skin Disease detection was evaluated using key metrics such as accuracy, precision, recall, F1-score, specificity, and AUC-ROC. These metrics were calculated based on the true positive (TP), true negative (TN), false positive (FP), and false negative (FN) values, providing a comprehensive assessment of each model’s effectiveness.

In Dataset 1, VGG16 emerged as the top performer, achieving the highest accuracy of 96.07%. This model also delivered outstanding precision, F1-score, and specificity. Notably, VGG19, ResNet152V2, MobileNetV2, and DenseNet201 achieved perfect precision and specificity, indicating their robustness in correctly identifying LSD cases without generating false positives. The Xception model, while slightly lower in accuracy, demonstrated the highest recall, making it particularly useful in scenarios where minimizing false negatives is crucial. The evaluation graphs illustrate the accuracy, loss, confusion matrix, and ROC curve for each model on Dataset 1, highlighting the strong performance of the top models and the relatively weaker performance of EfficientNetV2S, which had the lowest accuracy and recall.

For Dataset 2, MobileNetV2 achieved the highest accuracy at 96.39%, showing consistency and reliability across different datasets. ResNet152V2 and InceptionV3 also performed exceptionally well, particularly in terms of precision, achieving the highest values among all models. The F1-scores and recall values further supported the strong performance of these models, with MobileNetV2 and VGG16 leading in these metrics. Specificity, a critical metric for ensuring that healthy cattle are not misdiagnosed as having LSD, was maximized by ResNet152V2, InceptionV3, and NASNetMobile, each achieving near-perfect scores. The evaluation graphs for Dataset 2 reaffirm the superior performance of MobileNetV2 and ResNet152V2, while EfficientNetV2S again lagged behind, particularly in recall and F1-score, indicating potential limitations in generalizability.

In medical diagnostic processes, the sensitivity (also known as recall) of a model is crucial, particularly for contagious diseases like Lumpy Skin Disease (LSD). A high sensitivity indicates the model’s effectiveness in correctly identifying true positive cases, thereby minimizing false negatives. Reducing the number of false negatives is essential because a missed diagnosis can result in an infected cow not being isolated, leading to further spread of the disease within the herd. On Dataset 1, the Xception model achieved the highest sensitivity at 96.87%, closely followed by VGG16 with a sensitivity of 93.75%. The EfficientNetV2S model recorded the lowest sensitivity at 40.62%. The remaining models exhibited sensitivities ranging from 75% to 84.37%. For Dataset 2, VGG16 attained the highest sensitivity at 98.57%, making it the most effective model in identifying true positive cases of LSD in this dataset. MobileNetV2 followed with a sensitivity of 95.71%. Again, EfficientNetV2S had the lowest sensitivity at 41.42%, while the other models achieved sensitivities between 81.42% and 90%. These results highlight that VGG16 consistently delivers high sensitivity across different datasets, indicating its robustness and reliability in medical diagnostic applications. Its ability to accurately detect the majority of LSD cases makes it particularly suitable for early detection and intervention. High sensitivity is imperative in preventing the spread of LSD, as it ensures that infected cattle are promptly identified and isolated, thereby safeguarding the health of the entire herd.

The comparative analysis across both datasets highlights the strengths and weaknesses of each model. VGG16 and MobileNetV2 consistently performed well, making them reliable choices for LSD detection in varied datasets. ResNet152V2, with its high precision and specificity, is particularly advantageous for clinical settings where minimizing false positives is paramount. In contrast, EfficientNetV2S, despite its promising architecture, underperformed across most metrics in both datasets, suggesting that it may not be well-suited for LSD detection without further fine-tuning. The ROC curves and confusion matrices provide visual confirmation of these findings, showing clear distinctions in model performance, particularly in terms of sensitivity and specificity. This study demonstrates that deep learning models can significantly surpass traditional machine learning approaches like SVM and Naïve Bayes, which have shown lower classification accuracies in previous studies [28,29,30] (43.2% and 88.3%, respectively). The models tested here, particularly VGG16, MobileNetV2, and ResNet152V2, represent substantial advancements in the accurate and reliable detection of LSD, offering veterinarians powerful tools for early diagnosis and treatment.

### 3.10. Testing the Model’s Ability to Identify LSD in the Presence of Other Bovine Diseases

To evaluate the models’ ability to specifically identify Lumpy Skin Disease (LSD) amidst other bovine diseases, we created a new mixed dataset comprising two classes: Lumpy Skin and Non-Lumpy Skin. The Non-Lumpy Skin class included a total of 1432 images, consisting of the following:

- 545 images of healthy cows from [27];
- 44 images of cows suffering from dermatophilosis [28];
- 37 images of cattle with ringworm [28];
- 51 images of bovines suffering from pediculosis [28];
- 3 images of cows with both ringworm and pediculosis [28];
- 3 images of bovines with both dermatophilosis and pediculosis [28];
- 749 images of cows affected by foot and mouth disease (FMD) [27].

The Lumpy Skin class comprised 703 images of bovines affected by LSD. This dataset was specifically designed to assess how effectively the models can distinguish LSD from other skin diseases in cattle.

The top two models identified in this study, VGG16 and MobileNetV2, were trained and tested on this new dataset. VGG16 achieved an accuracy of 85.45%, with a precision of 76.71%, sensitivity of 80%, specificity of 88.11%, F1-score of 78.32%, and an AUC-ROC of 84.05%. In contrast, MobileNetV2 achieved a slightly higher accuracy of 85.91% but did not perform as well in terms of sensitivity, reaching only 57.14%. However, it attained a 100% precision and specificity, with an F1-score of 72.73% and an AUC-ROC of 78.57%. These results indicate that while MobileNetV2 is highly precise in correctly identifying LSD cases (no false positives), it is less sensitive and may miss true positive cases (higher false negatives). Conversely, VGG16 offers a more balanced performance between sensitivity and specificity, making it more suitable for practical diagnostic applications where detecting all true cases is critical.

### 3.11. Comparison of Proposed Model with State of the Art Models

To showcase the improvement in diagnostic performance provided by our proposed models, VGG16 and MobileNetV2, we compared them with other state-of-the-art models for LSD detection from previous studies. The comparison (Table 1 and Table 2) focuses on key performance metrics such as accuracy, precision, sensitivity, specificity, validation loss, and the number of trainable parameters. Table 1 compares the classification accuracy and validation loss of our proposed models with those reported in previous studies. It is evident that VGG16 and MobileNetV2 from our study surpass the accuracy of existing models. Additionally, our models achieve lower validation loss values, indicating better generalization and reliability. Table 2 presents a detailed comparison of additional performance metrics, including precision, sensitivity, specificity, and the number of trainable parameters. Our proposed models demonstrate superior precision and specificity compared to the models from prior studies. Notably, MobileNetV2 achieves a perfect precision and specificity of 100%, although its sensitivity is slightly lower than that of VGG16.

## 4. Discussion

The findings of this study underscore the significant potential of using transfer learning in veterinary science, particularly for the early detection and diagnosis of Lumpy Skin Disease in cattle. This research evaluates ten pre-trained deep learning models across two distinct datasets, demonstrating the practicality and efficiency of these models in a veterinary context. For veterinarians, the implications of these results are profound, offering new tools that can enhance diagnostic accuracy, speed, and overall animal health management.

### 4.1. Practical Implications for Veterinary Practice

From a veterinary perspective, the implementation of deep learning models like VGG16, MobileNetV2, and ResNet152V2 provides a robust framework for enhancing the diagnostic process. VGG16, which achieved the highest accuracy in this study, stands out for its reliability and ease of use. This model’s strong performance across datasets suggests that it can be a valuable tool in a veterinarian’s arsenal, particularly in field conditions where quick and accurate diagnosis is crucial. MobileNetV2’s success, particularly on the second dataset, highlights its suitability for resource-constrained environments—common in rural and remote veterinary practices. Designed for mobile applications, MobileNetV2 offers a high level of accuracy with lower computational demands, making it an ideal choice for veterinarians working in areas with limited access to advanced technological infrastructure. This model’s adaptability ensures that it can be deployed in various settings, from large-scale farms to small rural clinics, thereby expanding access to advanced diagnostic tools. ResNet152V2 and DenseNet201 also performed well, offering a balance between complexity and accuracy. These models are particularly useful for veterinarians dealing with more complex cases where detailed image analysis is required. ResNet’s ability to maintain high accuracy across different datasets makes it a reliable choice for veterinarians who need to ensure consistent results across diverse animal populations and environmental conditions.

### 4.2. Enhancing Diagnostic Accuracy and Speed

The ability to quickly and accurately diagnose LSD is critical for veterinarians, particularly given the contagious nature of the disease and its impact on livestock health and productivity. The high accuracy achieved by VGG16 and MobileNetV2 models in this study suggests that these tools can significantly reduce the time required for diagnosis, allowing for more immediate intervention. This is particularly important in managing outbreaks, where time is of the essence to prevent widespread transmission and economic loss. The models evaluated in this study provide veterinarians with the ability to detect even subtle signs of LSD that might be missed during a traditional visual inspection. This capability not only improves the accuracy of diagnoses but also enhances the overall quality of care provided to the animals. Early detection facilitated by these models can lead to more effective treatment plans, reducing the severity of outbreaks and improving recovery rates among affected cattle.

### 4.3. Integration into Veterinary Workflows

For these deep learning models to be effectively integrated into veterinary practice, it is essential to consider how they fit within existing workflows. The simplicity and efficiency of models like VGG16 and MobileNetV2 make them well-suited for integration into routine veterinary procedures. These models can be used alongside traditional diagnostic methods to provide a comprehensive assessment of an animal’s health, offering a second opinion that is both rapid and reliable. Moreover, the deployment of these models on mobile platforms enables veterinarians to carry out on-the-spot diagnostics, reducing the need for transporting animals to specialized facilities. This not only saves time and resources but also minimizes stress on the animals, leading to better overall welfare outcomes. By leveraging these technologies, veterinarians can enhance their diagnostic capabilities while maintaining a focus on animal welfare and care.

### 4.4. Addressing the Limitations and Ethical Considerations

While the results of this study are promising, it is important to recognize the limitations associated with the use of pre-trained models in veterinary practice. One of the key challenges is the variability in datasets, which may not fully represent the diversity of cattle populations in different regions. Future research should focus on expanding the range of datasets to include a wider variety of cattle breeds, ages, and environmental conditions. This will ensure that the models are robust and applicable across different contexts, enhancing their reliability in practice. Ethical considerations are also paramount when integrating AI into veterinary diagnostics. These models should be viewed as complementary tools that support, rather than replace, the expertise of veterinarians. The decision-making process should always involve the veterinarian’s judgment, with AI serving as an aid to enhance accuracy and efficiency. There is also a need to ensure that the use of AI does not lead to an over-reliance on technology, which could diminish the importance of traditional skills and knowledge in veterinary practice.

### 4.5. Future Directions for Veterinary Research

The results of this study open up several avenues for future research in veterinary science. One potential direction is the development of hybrid models that combine the strengths of multiple architectures, potentially leading to even greater accuracy and robustness in diagnostics. Additionally, research could explore the application of these models to other common livestock diseases, expanding their utility in veterinary practice. Another important area for future research is the exploration of AI’s role in enhancing the decision-making process in veterinary care. By integrating AI with other diagnostic tools and data sources, veterinarians can gain a more comprehensive understanding of an animal’s health, leading to more informed and effective treatment plans. This holistic approach could significantly improve outcomes in veterinary care, particularly in complex or resource-limited environments.

## 5. Conclusions

This study underscores the significant potential of deep learning models for the early detection of Lumpy Skin Disease in cattle, a critical need in the veterinary field given the disease’s rapid spread and severe economic impact. Among the ten pre-trained models evaluated, VGG16 and MobileNetV2 consistently demonstrated superior accuracy, precision, and generalizability across different datasets, making them particularly valuable for practical implementation in diverse veterinary settings. The findings highlight that while VGG16 excels in accuracy, MobileNetV2 offers a balanced performance with lower computational demands, ideal for resource-constrained environments. However, this study also reveals limitations, particularly with models like EfficientNetV2S, which, despite its advanced architecture, underperformed in this specific application. This suggests that not all state-of-the-art models are universally applicable, and careful consideration of the dataset and task-specific characteristics is crucial in model selection. The research contributes to the growing body of evidence supporting the integration of artificial intelligence in veterinary diagnostics, offering tools that enhance diagnostic accuracy and speed, thereby improving animal health outcomes. Future work should focus on expanding the diversity of training datasets to improve model robustness across different cattle breeds and environmental conditions. Additionally, there is a need for further exploration into hybrid models that combine the strengths of multiple architectures to optimize performance.

## Figures and Tables

**Table 1:** Comparison of classification accuracy and validation loss.

Model	Reference	Accuracy (%)	Validation Loss	Trainable Parameters
EfficientNetB7, MobileNetV2	[16]	90.00	0.26	-
ResNet-50	[22]	93.90	-	-
VGG16	[31]	87.00	-	-
VGG19	[31]	86.00	-	-
InceptionV3	[31]	85.00	-	
VGG16 (Proposed)	This study	96.07	0.1981	527,362
MobileNetV2 (Proposed)	This study	93.13	0.2299	1,313,794

**Table 2:** Detailed performance metrics comparison.

Model Parameters	Reference	Precision (%)	Sensitivity (%)	Specificity (%)	Trainable Parameters
DenseNet121	[25]	99.00	-	-	-
CNN	[32]	85.70	60.00	97.10	25,300,066
VGG16 (Proposed)	This study	93.75	93.75	97.14	527,362
MobileNetV2 (Proposed)	This study	99.90	78.12	99.90	1,313,794

## Data Availability

The data presented in this study are available on request from the corresponding author. The data is not publicly available due to proprietary restrictions.
